# Preoperative radiotherapy combined with 5 days per week capecitabine chemotherapy in locally advanced rectal cancer

**DOI:** 10.1038/sj.bjc.6604042

**Published:** 2007-11-06

**Authors:** I Craven, A Crellin, R Cooper, A Melcher, P Byrne, D Sebag-Montefiore

**Affiliations:** 1 Leeds Cancer Centre, Cookridge Hospital Lane, Leeds LS16 6QB, UK

**Keywords:** locally advanced rectal cancer, neoadjuvant chemoradiotherapy, capecitabine, circumferential resection margin

## Abstract

There is increasing evidence supporting the use of preoperative chemoradiotherapy in patients with locally advanced rectal cancer in an attempt to facilitate complete surgical resection with clear margins. We describe our experience of using a 5-day per week regime of preoperative capecitabine chemoradiotherapy. Between November 2004 and September 2006, 70 patients with MRI-defined locally advanced rectal cancer were selected for treatment. Capecitabine was given at a dose of 900 mg m^−2^ for 5 days per week combined with 45 Gy of radiotherapy in 25 doses. This regime was well tolerated with 89% of our patients receiving the full dose of chemotherapy and 96% receiving the full dose of radiotherapy. Ninety-three per cent proceeded to macroscopically complete surgical resection. The pathological complete response rate was 9.2% with a node-negative rate of 66%. A negative circumferential margin was achieved by 79% of the patients who underwent resection. Compared to studies using a 7-day per week capecitabine schedule, our results show increased compliance and less dose reductions with comparable pathological outcome.

There is clear evidence from two systematic reviews that adjuvant radiotherapy reduces the risk of local recurrence in patients with resectable rectal cancer (Camma *et al*, 2000; Colorectal Cancer Collaborative, 2001). The greatest benefit is seen when preoperative radiotherapy is used with a biologically equivalent dose of >30 Gy. Further clinical trials were required to assess the benefit of adding concurrent chemotherapy to preoperative radiotherapy.

The recent publication of two phase III trials confirms that preoperative concurrent chemoradiation (CRT) is superior to long-course radiotherapy alone in patients with T3/4 or node-positive resectable rectal cancer. The EORTC 22921 (Bosset *et al*, 2005) and FFCD 9203 (Gerard *et al*, 2006) trials compared intravenous 5-fluorouracil (5FU) and leucovorin (LV) given during the first and fifth weeks of radiotherapy with radiotherapy alone (dose of RT was 45 Gy in 25 fractions in both arms of the studies). Both trials demonstrated a reduction in the rates of local recurrence at 5 years from 17% with radiotherapy alone to 8–9% with preoperative CRT, but neither trial showed any difference in overall survival. A further recent phase III trial has demonstrated that preoperative 5FU CRT is superior to post-operative CRT with a significant reduction in local recurrence from 12 to 6% as well as a significant reduction in both acute and long-term toxicity (Sauer *et al*, 2004). This evidence will clearly result in an increasing use of fluoropyrimidine-based CRT.

Intravenous 5FU-based chemoradiation presents many logistical challenges. Continuous infusion of 5FU requires the insertion of a central venous line with its attendant risks of thrombosis and infection as well as the inconvenience of a portable infusion pump. The use of the bolus 5FU/LV regimen used in the EORTC and FFCD trials requires 10 daily visits to the chemotherapy day unit, with associated waiting time, the need for repeat venepuncture and the need for close coordination of the timing of the delivery of chemotherapy and radiation.

Capecitabine is an oral, tumour-activated fluoropyrimidine. Two large randomised phase III trials have compared capecitabine against low-dose LV and 5FU (Mayo regimen) in patients with metastatic colorectal cancer with no difference in time to progression or overall survival (Van Cutsem *et al*, 2004). In addition, two randomised, controlled trials have shown at least equivalent disease-free survival with the use of an oral fluropyrimidine when compared with low-dose LV and 5FU as post-operative adjuvant therapy for stages II and III colon cancer (Wolmark *et al*, 2004; Twelves *et al*, 2005). Furthermore, the use of oral capecitabine has been supported by recent NICE guidance on the management of colon cancer in both the metastatic and adjuvant settings (NICE, 2003, 2006). These results support the rationale for developing oral fluoropyrimidine-based chemoradiation.

Two formal dose-finding studies have been performed that escalate the dose of capecitabine when added to a fixed dose of pelvic radiation in rectal cancer. Dunst *et al* (2002) reported a recommended dose of 825 mg m^−2^ 7 days per week for phase II study, whereas Ngan *et al* (2004) recommended 900 mg m^−2^ 5 days per week. We decided to further evaluate the use of the latter regimen.

In the United Kingdom, high-resolution pelvic MRI is increasingly used to determine the relationship of the primary tumour to the potential margins of surgical resection. This correlation was reported by Brown *et al* (2003) in a single-centre experience and has been described by other centres (Blomqvist *et al*, 1999; Beets-Tan *et al*, 2001; Bissett *et al*, 2001; Botterill *et al*, 2001). A large international prospective multicentre study (MERCURY trial) has demonstrated that the extent of tumour seen on the preoperative MRI scan is equivalent to that seen in the resected pathological specimen in patients who undergo initial surgery with or without short-course preoperative radiotherapy (MERCURY Study Group, 2006). These findings led us to adopt the routine use of pelvic MRI in determining the selection of patients for preoperative radiotherapy and chemoradiation (Sasapu *et al*, 2006). Using pelvic MRI, patients are selected for preoperative CRT if the margins of resection are considered to be at risk. The main aim of treating such patients is to achieve a macroscopic complete resection with histologically confirmed clear (uninvolved) resection margins.

## PATIENTS AND METHODS

Between November 2004 and September 2006, patients with locally advanced rectal cancer were selected for preoperative capecitabine CRT on clinical and radiological findings. CRT was indicated if (i) the mesorectal fascia (MRF) was threatened (macroscopic tumour located within 2 mm of the MRF on the MRI), (ii) the MRF was involved (up to or beyond the MRF on the MRI), or (iii) there was T3 or T4 tumour with distal extent within 5 cm of the anal verge.

The patient was considered suitable for capecitabine therapy, if their glomerular filtration rate was >50 ml min^−1^ and they were able to comply with self-medication.

### Radiotherapy

Clinical examination determined the distal extent of the primary tumour, and the pelvic MR images were used to determine the gross tumour volume, which is outlined on individual slices of a computed tomography (CT) planning scan. Margins were added to the gross tumour volume to create the planning target volume (3 cm lateral and 3–5 cm superior, 2 cm anterior and 2–3 cm inferior). The posterior border was always located at the posterior border of the body of the sacrum. Unless contraindicated, patients were treated in the prone position with a full bladder using a 3- or 4-field technique. Small bowel imaging was achieved using oral gastrograffin (20 ml in 1000 ml water) 45 minutes prior to the planning CT scan. A total dose of 45 Gy in 25 fractions over 5 weeks was prescribed to the ICRU (intersection) point and this was delivered using 3- or 4-radiation beams with the use of multileaf collimation.

### Chemotherapy

Capecitabine was prescribed at a dose of 900 mg m^−2^ twice daily for 5 days per week for 5 weeks. The patients were advised to take the prescribed capecitabine dose at 0900 and 2100 hours on the days they received radiotherapy. Routine haematological tests (full blood count and urea and electrolytes) were carried out before CRT commenced and again at week 3. The patients were monitored weekly for toxicities. In the presence of grade III/IV acute toxicity, the dose of capecitabine was reduced or if necessary discontinued.

### Surgery and histopathology

Prior to surgery, patients were reassessed 4–6 weeks after completion of CRT by repeat pelvic MRI±thoracoabdominal CT. The recommended timing to perform surgery was 6–8 weeks following CRT.

The resected specimen was examined using the methods described by Quirke *et al* (1986). The tumour was not opened and was fixed in formalin. The circumferential margin was then stained with Indian ink and axial thin slices were made. The specimen was examined macroscopically and blocks were taken from all suspicious areas. Pathological complete response (pCR) was defined by the complete absence of tumour cells from the entire specimen. The circumferential resection margin (CRM) was routinely evaluated and considered to be involved if there was evidence of any microscopic tumour ⩽1 mm from the painted CRM edge.

## RESULTS

Between November 2004 and September 2006, 70 patients from the Yorkshire Cancer Network (45 men and 25 women) have been selected for capecitabine CRT. The tumour characteristics at preoperative staging are shown in Table 1. Thirty-two patients (46%) had tumours with a distal limit <5 cm from the anal verge. Thirty-four patients (49%) had tumours >5 cm from the anal verge (31 patients 5–10 cm and 3 patients >10 cm) and there were four patients, where the distance was not stated. Of the 34 patients with mid to upper rectal tumours (>5 cm from anal verge), 22 had a threatened MRF (65%) and 12 had tumour involving the MRF (35%).

### Acute toxicity and compliance

All 70 patients have completed their treatment and were assessed for capecitabine CRT toxicity. Sixty-two (88.6%) patients received the full dose of capecitabine without suffering significant grade 3 or 4 toxicities. Three (4.3%) patients had to discontinue capecitabine due to acute toxicities. One patient with a known cardiac history developed unstable angina and was admitted to coronary care unit; one patient had pre-existing ulcerative colitis and was operated on acutely for large bowel distension; one patient developed severe abdominal pain and diarrhoea. It is not possible to determine the relative contribution of the capecitabine and radiotherapy in the cause of the abdominal toxicity.

Five (7.1%) patients required dose reductions in capecitabine. One patient had a low glomerular filtration rate at commencement of therapy and received an elective 20% dose reduction throughout treatment; one patient suffered from a deterioration in renal function at week 3 (creatinine 129 from 89 *μ*mol l^−1^ prior to treatment commencing), and the dose was omitted for a week and then started at a 20% reduction; three patients suffered grade 3 diarrhoea and had dose reductions of between 10 and 25%.

Sixty-seven (96%) patients received the full 45-Gy dose of radiotherapy. Out of the three patients (4%) who failed to receive the full dose, one patient was considered too unwell to receive his last dose (received 43.2 Gy in total), and two patients were admitted with abdominal pain (described above) having received 41.4 and 37.8 Gy, respectively. Two other patients required a break in RT (2–5 days) due to capecitabine toxicity.

### Surgical resection

Complete macroscopic resection was performed in 65 of the 70 patients (93%) who received capecitabine CRT. Resection was not performed in five patients. Two patients developed inoperable lung metastases identified on the CT scan performed 6 weeks after completion of CRT; one patient died from disease progression shortly after CRT had finished; one patient developed inoperable disease with sacral involvement; one patient was not resected because of an unacceptably high risk of perioperative mortality due to pre-existing cardiorespiratory comorbidities.

One patient had an emergency resection 9 days after completing CRT for large bowel obstruction. The timing of elective surgical resection varied from weeks 4 to 23 post-CRT, with a median of 8 weeks (interquartile range: 7–9.5 weeks).

### Histopathology

The histopathological results are summarised in Table 2. Of the 65 patients who underwent resection, 6 patients (9.2%) had a complete pathological response (pCR or ypT_0_N_0_). One further patient had complete regression of the primary tumour but had 1 out of 22 positive lymph nodes (ypT_0_N_1_). There were 21 (32.3%) patients with ypT_0–2_ tumours. Out of the 64 patients, where preoperative MRI staging was available, 26 patients (41%) were downstaged by CRT and in 4 patients (6%) disease progressed. Twenty-two (33.8%) patients had node-positive disease. The median number of nodes examined was 13.2 (interquartile range: 7–18).

The number of resections achieving a negative CRM was high with 51 (79.7%) specimens having an uninvolved CRM (one specimen could not be evaluated due to surgical trauma). Table 3 shows the CRM status by operation performed and tumour location. Circumferential resection margin involvement was seen in 15.2% of anterior resections (5 out of 33) compared to 7 out of 26 abdominoperineal excision resection specimens (26.9%). The rate of CRM involvement for tumours within 5 cm of the anal verge was 29% (9 out of 31), whereas it was 13.3% (4 out of 30) in higher tumours (>5 cm).

## DISCUSSION

This series of patients, receiving a 5-day per week schedule of capecitabine and radiotherapy, shows similar efficacy and high compliance compared to intravenous 5FU/LV regimes. In this series, 67 (96%) patients received the full 45 Gy dose of radiotherapy and this is comparable to the large EORTC (96%) and FFCD (97.1%) trials. Sixty-two patients (89%) received the protocol dose of capecitabine compared to 83.7% receiving the correct dose of i.v. 5FU/LV in the EORTC and 78.1% in the FFCD trials. In our study, a further 7% of patients completed the course of capecitabine with a 10–25% dose reduction.

At the time of writing, there were two published phase II trials of capecitabine CRT (De Paoli, 2006; Krishnan *et al*, 2006) and a further study comparing capecitabine CRT to 5FU/LV (Kim *et al*, 2007), and all three used the 825 mg m^−2^ continuous daily schedule. In these studies, the number of patients receiving the full dose of capecitabine was much lower compared to our 5-day schedule. The study by De Paoli showed that 72% of patients received the full dose, whereas in the study by Krishnan, 11 out of the 54 (20%) patients received a dose reduction, and 11 out of the 54 (20%) patients had treatment stopped before completion (it is not clear whether or not these were the same 11 patients). The study by Kim *et al* (2007) published no data on the number of patients receiving dose reductions but quoted a 99.3% chemotherapy completion rate. Without detailed toxicity data, it is difficult to compare compliance between the 5- and 7-day regimes, but the available evidence suggests that more dose modifications are required using a 7-day schedule.

In our study, 93% of the patients who received capecitabine CRT went on to have a complete macroscopic resection, although there is some variation in the timing of surgery. It was recommended that surgery took place 6–8 weeks following completion of CRT, although most colorectal multi-disciplinary teams (MDTs) accept that the optimised timing of surgery is not defined. We feel that the quoted interquartile range of 7.5–9 weeks demonstrates acceptable compliance with our recommendations. Over 50% of the patients had surgery 6–8 weeks, and 79% had surgery 6–10 weeks following completion of CRT. The delays in surgery appear to be simply due to the challenge of finding available operating time. There is no evidence to suggest that surgery was delayed to allow more time for tumour regression.

There was only one patient who had a resection before 4 weeks due to developing abdominal signs and symptoms requiring urgent surgery. Of the remaining 64 patients, 30 had surgery >8 weeks and 34 had surgery ⩽8 weeks. The pathological ypT0-2 stage was 40 and 23%, the node-negative rate was 71 and 60% and the CRM-negative rate was 86 and 73%, respectively. An APER was performed in 13 out of the 35 (37%) patients who underwent surgery ⩽8 weeks and 14 out of the 30 (47%) patients who underwent surgery >8 weeks after completion of CRT. The numbers are too small to allow formal statistical comparison.

It is important to emphasise that this group of patients were defined by MRI as locally advanced, where the margin of excision was considered at risk. Of the 66 patients classified preoperatively, 11 (17%) presented with T4 and 52 (79%) presented with T3 disease on pelvic MRI. The tumour was directly threatening or involving the resection margins in 61 cases (88%). Due to this caseload, we may expect to see lower rates of downstaging compared to other studies. The pCR rate in this study was 9.2% (EORTC 13.7%, FFCD 12.1%), with an additional patient having a complete response of the primary tumour but positive nodes (1 out of 22 nodes were positive). The rate of ypT_0–2_ tumours was 32.3% with 41% of patients downstaged, according to their diagnostic MRI. Due to the advanced nature of presentation one would expect a high rate of lymph node involvement, so a node-negative rate of 66.2% is also encouraging.

**Figure 1:**
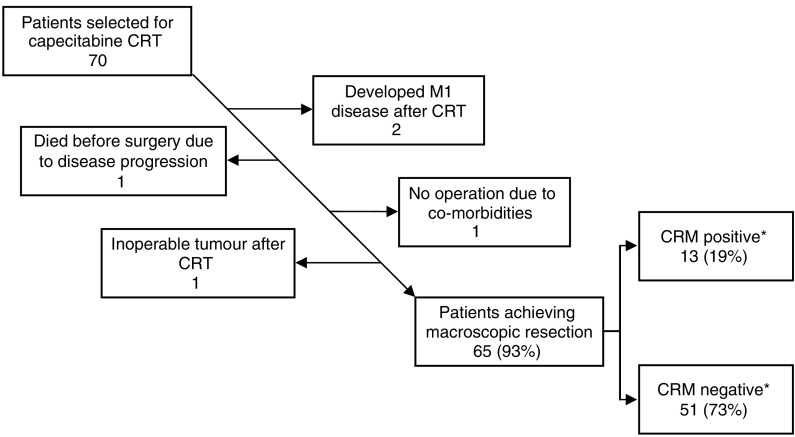
Outcome of the 70 patients selected for capecitabine chemoradiotherapy (^*^one specimen was not assessed for CRM status due to surgical trauma).

The number of resections achieving a negative CRM status is high (79.7%). These results compare favourably with pooled data from 677 patients treated in six UK centres with 5FU/LV pre-op CRT, where the pCR rate was 13%, and 388 (70%) of resected patients achieved an uninvolved CRM (Sebag-Montefiore *et al*, 2005b). The intention-to-treat analysis (see Figure 1) shows that we achieved a negative CRM in 51 out of the 70 cases (73%). This compares favourably to the 65% CRM-negative rate reported in a study using 5FU/LV in patients presenting with similar advanced disease (Mawdsley *et al*, 2005). The ITT pCR rate (i.e., from the total number of patients selected for CRT) was similar in both studies (8.6% in our study *vs* 10% in the study by Mawdsley).

The value of using the degree of response to CRT (either pCR or rate of downstaging) as an end point for assessing efficacy is debatable. Circumferential resection margin status predicts for local recurrence and overall survival (Birbeck *et al*, 2002; Wibe *et al*, 2002). However, CRM status is rarely reported. In a review of 215 phase II and III studies of radiotherapy and chemoradiotherapy in rectal cancer, only 14 presented data on the negative CRM rate (Glynne-Jones *et al*, 2006).

Table 3 shows the CRM status for different operations and different levels of tumour. There is a higher rate of CRM involvement in patients with low tumours and this is seen for both tumour position (<5 cm from the anal verge – 29%) and by operation performed (abdominoperineal resection – 25.9%). The overall rate of CRM involvement (20.3%) remains high and emphasises the need to improve treatment either by intensifying the preoperative CRT or by improving surgical techniques.

The argument for intensifying CRT is supported by a systematic analysis of phase II and III trials in preoperative CRT in rectal cancer that suggested that the use of a second chemotherapeutic agent increased the pCR rate (*P*=0.001). This has been supported by several large phase II trials (Glynne-Jones *et al*, 2005, 2007; Sebag-Montefiore *et al*, 2005a; Gollins *et al*, 2006).

Abdominoperineal resections are associated with increased CRM involvement, increased intraoperative tumour perforations and decreased overall survival (Marr *et al*, 2005; Nagtegaal *et al*, 2005). An alternative approach to the perineal element of the abdominoperineal resection, designed to minimise circumferential involvement, has been proposed with promising initial results (Holm *et al*, 2007).

This is the only large study of a 5-day per week capecitabine schedule. This series of patients shows that this regimen has excellent compliance and tolerance, with similar efficacy to other studies published and could be used in future phase III trials, looking at the addition of a second chemotherapy drug or antibody. It would be particularly useful to have a 5-day per week capecitabine CRT schedule if the novel arm in a phase III trial used a similar schedule (e.g., the CORE regimen using oxaliplatin 50 mg m^−2^ once weekly and capecitabine 825 mg m^−2^ b.d. 5 days per week, both for 5 weeks).

## Figures and Tables

**Table 1:** Tumour characteristics on presentation and the toxicities observed during capecitabine CRT

	**Number of patients**		
**Tumour characteristics**	**Total (%)**	**Male**	**Female**
Number of patients	70	45	25
			
*Distance from anal verge* (*cm*)			
0–5	32 (46)	19	13
5–10	31 (44)	22	9
10–15	3 (4)	3	0
Unknown	4 (6)	1	3
			
*Pre*-*op staging* (*MRI*)			
T2	3 (4)	1	2
T3	52 (74)	34	18
T4	11 (16)	8	3
Not stated	4 (6)	2	2
			
*Compliance with treatment*			
*Capecitabine therapy*			
Full dose	62 (89)	44	18
Dose reduction	5 (7)	0	5
Did not complete course	3 (4)	1	2
			
*Radiotherapy*			
Full dose	67^a^ (96)	43	24^a^
Did not complete course	3 (4)	2	1

CRT=concurrent chemoradiation; MRI=magnetic resonance imaging.

a Two patients received the full dose of radiotherapy but required a break from treatment of between 2 and 5 days due to capecitabine toxicity.

**Table 2:** Pathological stages of the patients who underwent surgical resection^a^

	**ypT stage**					**ypN stage**	
**Pre-op MRI**	**ypT0**	**ypT1**	**ypT2**	**ypT3**	**ypT4**	**ypN0**	**ypN+**
Not stated			1	1	1	2	1
T2	1		1			1	1
T3	5	1	10	30	4	31	19
T4	1		1	7	1	9	1
							
Total (%)	7 (11%)	1 (1.5%)	13 (20%)	38 (59%)	6 (9%)	43 (66%)	22 (34%)

MRI=magnetic resonance imaging.

a The circumferential resection margin was not assessed on one specimen due to surgical trauma.

**Table 3:** The rates of CRM positivity in different groups of patients

		**CRM status**		**CRM-positive rate**
	**Total**	**Positive**	**Negative**	**(%)**
Whole population^a^	64	13	51	20.3
				
*Operation*				
Anterior resection	33	5	28	15.2
Abdominoperineal resection	26	7	19	26.9
Pelvic exenteration	5	1	4	20.0
				
*Distance from anal verge*^b^ (*cm*)				
0–5	31	9	22	29.0
>5	30	4	26	13.3

CRM=circumferential resection margin.

a One patient who had an abdominoperineal resection and a tumour 4 cm from the anal verge was not assessed due to trauma to the specimen.

b Three patients with unknown tumour location.

## References

[bib1] Beets-Tan RG, Beets GL, Vliegen RF, Kessels AG, Van Boven H, De Bruine A, von Meyenfeldt MF, Baeten CG, van Engelshoven JM (2001) Accuracy of magnetic resonance imaging in prediction of tumour-free resection margin in rectal cancer surgery [see comment]. Lancet 357: 497–504 11229667 10.1016/s0140-6736(00)04040-x

[bib2] Birbeck KF, Macklin CP, Tiffin NJ, Parsons W, Dixon MF, Mapstone NP, Abbott CR, Scott N, Finan PJ, Johnston D, Quirke P (2002) Rates of circumferential resection margin involvement vary between surgeons and predict outcomes in rectal cancer surgery. An Surg 235: 449–457 10.1097/00000658-200204000-00001 PMC1422458 11923599

[bib3] Bissett IP, Fernando CC, Hough DM, Cowan BR, Chau KY, Young AA, Parry BR, Hill GL (2001) Identification of the fascia propria by magnetic resonance imaging and its relevance to preoperative assessment of rectal cancer. Dis Colon Rectum 44: 259–265 11227944 10.1007/BF02234302

[bib4] Blomqvist L, Rubio C, Holm T, Machado M, Hindmarsh T (1999) Rectal adenocarcinoma: assessment of tumour involvement of the lateral resection margin by MRI of resected specimen. Br J Radiol 72: 18–23 10341684 10.1259/bjr.72.853.10341684

[bib5] Bosset J-F, Calais G, Mineur L, Maingon P, Radosevic-Jelic L, Daban A, Bardet E, Beny A, Briffaux A, Coillette L (2005) Enhanced tumoricidal effect of chemotherapy with preoperative radiotherapy for rectal cancer: preliminary results – EORTC 22921. J Clin Oncol 23: 5620–5627 16009958 10.1200/JCO.2005.02.113

[bib6] Botterill ID, Blunt DM, Quirke P, Sebag-Montefiore D, Sagar PM, Finan PJ, Chalmers AG (2001) Evaluation of the role of pre-operative magnetic resonance imaging in the management of rectal cancer. Colorectal Dis 3: 295–303 12790949 10.1046/j.1463-1318.2001.00258.x

[bib7] Brown G, Radcliffe AG, Newcombe RG, Dallimore NS, Bourne MW, Williams GT (2003) Preoperative assessment of prognostic factors in rectal cancer using high-resolution magnetic resonance imaging. Br J Surg 90: 355–364 12594673 10.1002/bjs.4034

[bib8] Camma C, Giunta M, Fiorica F, Pagliaro L, Craxi A, Cottone M (2000) Preoperative radiotherapy for resectable rectal cancer: a meta-analysis. JAMA 284: 1008–1015 10944647 10.1001/jama.284.8.1008

[bib9] Colorectal Cancer Collaborative Group (2001) Adjuvant radiotherapy for rectal cancer: a systematic overview of 8507 patients from 22 randomised trials [see comment]. Lancet 358: 1291–1304 11684209 10.1016/S0140-6736(01)06409-1

[bib10] De Paoli A (2006) Capecitabine in combination with preoperative radiation therapy in locally advanced, resectable, rectal cancer: a multicentric phase II study. Ann Oncol 17: 246–251 16282246 10.1093/annonc/mdj041

[bib11] Dunst J, Reese T, Sutter T, Zuhlke H, Hinke A, Kolling-Schlebusch K, Frings S (2002) Phase I trial evaluating the concurrent combination of radiotherapy and capecitabine in rectal cancer. J Clin Oncol 20: 3983–3991 12351595 10.1200/JCO.2002.02.049

[bib12] Gerard J-P, Conroy T, Bonnetain F, Bouche O, Chapet O, Closon-Dejardin M, Untereiner M, Leduc B, Francois E, Maurel J, Seitz J-F, Buecher B, Mackiewicz R, Ducreux M, Bedenne L (2006) Preoperative radiotherapy with or without concurrent fluorouracil and leucovorin in T3-4 rectal cancers: results of FFCD 9203. J Clin Oncol 24: 4620–4625 17008704 10.1200/JCO.2006.06.7629

[bib13] Glynne-Jones R, Falk S, Maughan TS, Meadows HM, Sebag-Montefiore D (2007) A phase I/II study of irinotecan when added to 5-fluorouracil and leucovorin and pelvic radiation in locally advanced rectal cancer: a Colorectal Clinical Oncology Group Study. Br J Cancer 96: 551–558 17262086 10.1038/sj.bjc.6603570 PMC2360056

[bib14] Glynne-Jones R, Mawdsley S, Novell JR (2006) The clinical significance of the circumferential resection margin following preoperative pelvic chemo-radiotherapy in rectal cancer: why we need a common language. Colorectal Dis 8: 800–807 17032329 10.1111/j.1463-1318.2006.01139.x

[bib15] Glynne-Jones R, Sebag-Montefiore D, Samuel L, Falk S, Maughan TS, McDonald A (2005) Socrates phase II study results: capecitabine (CAP) combined with oxaliplatin (OX) and preoperative radiation (RT) in patients (pts) with locally advanced rectal cancer (LARC). J Clin Oncol 23, Abstract 3527

[bib16] Gollins SW, Myint S, Levine E, Bishop J, Haylock B, Susnerwala S, Saunders M, Biswas A (2006) Radiotherapy plus concurrent irinotecan (CPT-11) and capecitabine (CAP) as preoperative downstaging treatment for locally advanced inoperable rectal cancer: a phase I/II study. J Clin Oncol 24, Abstract 13519

[bib17] Holm T, Ljung A, Haggmark T, Jurell G, Lagergren J (2007) Extended abdominoperineal resection with gluteus maximus flap reconstruction of the pelvic floor for rectal cancer. Br J Surg 94: 232–238 17143848 10.1002/bjs.5489

[bib18] Kim DY, Jung KH, Kim TH, Kim DW, Chang HJ, Jeong JY, Kim YH, Son SH, Yun T, Hong CW, Sohn DK, Lim SB, Choi HS, Jeong SY, Park JG (2007) Comparison of 5-fluorouracil/leucovorin and capecitabine in preoperative chemoradiotherapy for locally advanced rectal cancer. Int J Radiat Oncol Biol Phys 67: 378–384 17097835 10.1016/j.ijrobp.2006.08.063

[bib19] Krishnan S, Janjan NA, Skibber JM, Rodriguez-Bigas MA, Wolff RA, Das P, Delclos ME, Chang GJ, Hoff PM, Eng C, Brown TD, Crane CH, Feig BW, Morris J, Vadhan-Raj S, Hamilton SR, Lin EH (2006) Phase II study of capecitabine (Xeloda) and concomitant boost radiotherapy in patients with locally advanced rectal cancer. Int J Radiat Oncol BiolPhys 66: 762–771 10.1016/j.ijrobp.2006.05.063 17011451

[bib20] Marr R, Birbeck K, Garvican J, Macklin CP, Tiffin NJ, Parsons WJ, Dixon MF, Mapstone NP, Sebag-Montefiore D, Scott N, Johnston D, Sagar P, Finan P, Quirke P (2005) The modern abdominoperineal excision: the next challenge after total mesorectal excision. Ann Surg 242: 74–82 15973104 10.1097/01.sla.0000167926.60908.15 PMC1357707

[bib21] Mawdsley S, Glynne-Jones R, Grainger J, Richman P, Makris A, Harrison M, Ashford R, Harrison RA, Osborne M, Livingstone JI (2005) Can histopathologic assessment of circumferential margin after preoperative pelvic chemoradiotherapy for T3–T4 rectal cancer predict for 3-year disease-free survival? Int J Radiat Oncol Biol Phys 63: 745–752 16199310 10.1016/j.ijrobp.2005.03.003

[bib22] MERCURY Study Group (2006) Diagnostic accuracy of preoperative magnetic resonance imaging in predicting curative resection of rectal cancer: prospective observational study. BMJ 333: 779–782 16984925 10.1136/bmj.38937.646400.55 PMC1602032

[bib23] Nagtegaal ID, van de Velde CJ, Marijnen CA, van Krieken JH, Quirke P (2005) Low rectal cancer: a call for a change of approach in abdominoperineal resection. J Clin Oncol 23: 9257–9264 16361623 10.1200/JCO.2005.02.9231

[bib24] Ngan SYK, Michael M, Mackay J, McKendrick J, Leong T, Lim Joon D, Zalcberg JR (2004) A phase I trial of preoperative radiotherapy and capecitabine for locally advanced, potentially resectable rectal cancer. Br J Cancer 91: 1019–1024 15305186 10.1038/sj.bjc.6602106 PMC2747703

[bib25] NICE (2003) Guidance on the use of capecitabine and tegafur with uracil for metastatic colorectal cancer. In: NICE Technology Appraisal 61, National Institute of Clinical Excellence

[bib26] NICE (2006) Capecitabine and oxaliplatin in the adjuvant treatment of stage III (Duke's C) colon cancer. In: Technology Appraisal 100, National Institute for Health and Clinical Excellence

[bib27] Quirke P, Dixon MF, Durdey P, Williams NS (1986) Local recurrence of rectal adenocarcinoma due to inadequate surgical resection: histopathological study of lateral tumour spread and surgical excision. Lancet 328: 996–999 10.1016/s0140-6736(86)92612-7 2430152

[bib28] Sasapu KK, Sebag-Montefiore D, Chalmers AG, Sagar PM, Burke D, Finan PJ (2006) Evaluation of a protocol-based management of rectal cancer. Dis Colon Rectum 49: 1703–1709 17028915 10.1007/s10350-006-0682-3

[bib29] Sauer R, Becker H, Hohenberger W, Rodel C, Wittekind C, Fietkau R, Martus P, Tschmelitsch J, Hager E, Hess CF, Karstens J-H, Liersch T, Schmidberger H, Raab R (2004) Preoperative *vs* postoperative chemoradiotherapy for rectal cancer. N Engl J Med 351: 1731–1740 15496622 10.1056/NEJMoa040694

[bib30] Sebag-Montefiore D, Brown G, Rutten HJ, Rullier E, Peeters M, Glynne-Jones R, Van Cutsem E, Ricci S, Van de Velde C, Quirke P (2005a) An international phase II study of Capecitabine, Oxaliplatin, Radiotherapy and Excision (CORE) in patients with MRI-defined locally advanced rectal adenocarcinoma. Interim results. Eur J Cancer Supplements 3: 170

[bib31] Sebag-Montefiore D, Glynne-Jones R, Mortensen NJ, Bedi C, Wilson C, Geh JI, McDonald A (2005b) Pooled analysis of outcome measures including the histopathological R0 resection rate after pre-operative chemoradiation for locally advanced rectal cancer. Colorectal Dis 7, Abstract 20

[bib32] Twelves C, Wong A, Nowacki MP, Abt M, Burris 3rd H, Carrato A, Cassidy J, Cervantes A, Fagerberg J, Georgoulias V, Husseini F, Jodrell D, Koralewski P, Kröning H, Maroun J, Marschner N, McKendrick J, Pawlicki M, Rosso R, Schüller J, Seitz JF, Stabuc B, Tujakowski J, Van Hazel G, Zaluski J, Scheithauer W (2005) Capecitabine as adjuvant treatment for stage III colon cancer. N Engl J Med 352(26): 2696–2704 15987918 10.1056/NEJMoa043116

[bib33] Van Cutsem E, Hoff PM, Harper P, Bukowski RM, Cunningham D, Dufour P, Graeven U, Lokich J, Madajewicz S, Maroun JA, Marshall JL, Mitchell EP, Perez-Manga G, Rougier P, Schmiegel W, Schoelmerich J, Sobrero A, Schilsky RL (2004) Oral capecitabine *vs* intravenous 5-fluorouracil and leucovorin: integrated efficacy data and novel analyses from two large, randomised, phase III trials. Br J Cancer 90: 1190–1197 15026800 10.1038/sj.bjc.6601676 PMC2409640

[bib34] Wibe A, Rendedal PR, Svensson E, Norstein J, Eide TJ, Myrvold HE, Soreide O (2002) Prognostic significance of the circumferential resection margin following total mesorectal excision for rectal cancer. Br J Surg 89: 327–334 11872058 10.1046/j.0007-1323.2001.02024.x

[bib35] Wolmark N, Wieand B, Lembersky L, Colangelo R, Smith R, Pazdur R (2004) A phase III trial comparing oral UFT to FULV in stage II and III carcinoma of the colon: results of NSABP Protocol C-06. J Clini Oncol, 2004 ASCO Annual Meeting Proceedings (Post-Meeting Edn), vol. 22, (July 15 supplement), no. 14S, abstract 3508

